# A Plea for Beard

**Published:** 1861-01

**Authors:** 


### A PLEA FOR BEARD.

The evidences of human progress are displayed in the reformation of abuses and the avoidance of accustomed errors, as well as in making discoveries, or in striking out new paths in the ways of science. One of these evidences, among the male part of humanity at least, is the avoidance of those extreme caprices of fashion by which the beauty and simplicity of nature is substituted, and the human form disfigured. Men in enlightened nations no longer shave off their own hair and put on their heads the hair of another, and then dust it thickly over with white powder. The waist is no longer laced to an effeminate attenuation, and tinseled trappings and ruffles are seen no more. The distortion and disfigurement of nature is now left to the anthropophagist who tattoos his skin, to the celestial who produces an artificial talipes, and to the savage whose lip, ear, or nose is slit, or whose skull is flattened to his taste by compression.

Society is beginning to think that the designs of nature in the formation of the human body are with an object for our welfare, and most in accordance with true beauty and harmony.

In recent times, a single practice is the only interference with the evident designs of nature which customs of gentility tolerate. This is one of comparatively modern invention, and as an adopted habit, dates no more remotely than to the coronation of Louis XIII. of France, who ascended the throne at the age of nine years, and in adulation to whose beardless face his courtiers commenced the foppish practice of *shaving*. Since that time the fashion of closely shaving the face has, like other fashions, been repeatedly abandoned and revived.

A bearded cycle is now upon us. The razor is becoming an obsolete instrument, and much of the barber's "occupation 's gone." We can not determine whether this almost universal wearing of beard has been adopted as a mere caprice of fashion, a rational change in taste, a time and labor-saving design suited to a fast age, or as an intelligent appreciciation of its hygienic advantages.

Be it as it may, the practice has been for years gaining among the refined and intelligent classes all over the world, and much in every view of the matter can be claimed for it, while nought but the fogyish partiality for old usages can be argued against it.

The wearing of the entire beard has become general in Europe, and even the English, heretofore the most closely shaven of all nations, has at last adopted the "valanced" face.

Of beard as a fashion, or of its "formal cut," we are unconcerned, and care not whether the moustache be in imperial loyalty turned up and twisted at its points, or, in the style of the radical, turned obstinately down, but we desire to repeat some ascertained facts in the hygiene of beard.

Dr. Agnew says: "There are many reasons for believing that the beard should not be removed. In cold localities it is an important defense. The skin of the cheek is delicate, and is underlaid by a network of both motor and sensory nerves of the fifth and seventh pairs, which are peculiarly impressible by the influence of low temperature. Almost every one has felt the stiff and paralyzed condition of the face when, unprotected, it has been exposed to the frosty air of a winter day, and also the chattering teeth. Both toothache and neuralgia have been entirely removed by allowing the beard to grow. So in warm situations, it serves as a defense against the irritating effects of the sun's rays, preventing tan, freckles, inflammation and desquamation; thus it is very common to find boatmen, and those employed about the water, cultivate a luxuriant growth of beard to obviate these inconveniences.

"The moustache is well situated, not only to defend the lip, but to arrest and entangle particles of dust which would otherwise be drawn into the respiratory passages.

"Mr. Chadwick asserts, that travelers in Syria and Egypt suffer so much from the small particles of sand which fill the air, that they really find it necessary to delay the wilderness journey until their mustaches have grown. To all, therefore, whose occupations expose them to dust, such as threshers, millers, smiths, etc., the preservation of the growth will be useful."

The London *Lancet* has taken a decided stand in favor of bearded faces; and, in an article censuring a military officer for enforcing the soldiers under his command to shave, makes the following proper remarks:

"Nature has ordained that the face of a man shall be protected in certain parts by a hairy covering. Be it for use, be it for ornament, or be it for both, there it is in the form of beard, mustaches and whiskers. These constitute as necessary a structural appendage to a man as the mane to the horse and lion, the antlers to a stag, the horns to the ox, or the bristly whiskers to old grimalkin. The growth and perfection of these appendages are closely associated with strength, masculine power and virility, and although often regarded as supplementary organs, they indubitably severally owe their origin to a *necessity* of the animal frame. With the facial hirsute appendages of man important authorities have fallen out. The former appear worse than useless to them; they are positive nuisances, so let them be abolished! Nature shall be corrected for the mistake she has made by witnessing her best-formed and strongest specimens of humanity rubbed well over with yellow soap every morning, and scraped with a razor. A red, glazy, pimply chin, raw nostrils, hoarse voice, sore throat and feminine or 'lily-livered' appearance, shall henceforth grace the stalwart forms of our policemen and soldiers! We hope that science and common sense will come to the rescue, and not only let soldiers and policemen continue to wear upon their fac s the natural covering they have been given, but induce wheezing, sneezing, sore-throated, shivering mortals, who have hitherto trembled more at the keen edge of a January air or March wind than of a razor, to cease wasting their time at their ridiculous matutinal operation, and face their fellow-mortals like *men.* Bichat long ago asserted, that although numerous causes might exist to produce debility of the system in coincidence with the presence of beard, yet the general impression must be that there exists 'uncertain rapport entre elle [la barbe] et les forces.' It is probable, says the great physiologist, that the muscular energy is, up to a certain point, connected with the beard, and that this energy always diminishes a little when a man deprives himself of that appendage. * * * * The well known traveler, Mr. St. John, says that Walter Savage Landor was a great sufferer from sore throat for many years of his life, and that he lost the morbid disposition only by following the advice of the surgeon of the Grand Duke of Tuscany, viz: to let his beard grow. According to Mr. Chadwick, the sappers and miners of the French army, who are remarkable for the size and beauty of their beards, enjoy a special immunity from bronchial affections. What can be more absurd than to compel men exposed to all kinds of climatorial severities and changes, as are soldiers and policemen, to rub a strong alkali daily upon their faces, and then scrape the latter over with a sharp instrument! * * * * In our opinion, it would be a far more rational procedure, upon the part of people in authority, to compel those in their power to allow their hair to grow, than to make them shave it off. This much might, at any rate, be said in favor of the one, that it must be for the grower's own benefit, since he followed Nature; while all that could be legitimately adduced to support the other must be, that it pleased the commander's very queer fancy. As regards certain public servants, too, like soldiers and policemen, the right which we have to enforce a particular garb or uniform upon them, and the necessity which exists for so doing, do not extend to tattooing them, nor even to that simpler mutilation—shaving—which is akin to it, seeing that it entails the loss of an important structural appendage bestowed upon them by Nature."

